# High-risk human papillomavirus infection and associated factors in the anal canal of HIV-positive patients in Medellín, 2017–2018

**DOI:** 10.11606/s1518-8787.2020054001692

**Published:** 2020-10-23

**Authors:** Daniela Herrera Posada, Lucia Stella Tamayo Acevedo, Marleny Valencia Arredondo, Gloria Inéz Sánchez Vásquez

**Affiliations:** I Universidad de Antioquia Escuela de Microbiología MedellínAntioquia Colombia Universidad de Antioquia . Escuela de Microbiología . Medellín , Antioquia , Colombia; II Universidad de Antioquia Facultad de Medicina MedellínAntioquia Colombia Universidad de Antioquia . Facultad de Medicina . Medellín , Antioquia , Colombia

**Keywords:** Human Papillomavirus Infections, Epidemiology, HIV Infections, Anal Neoplasia, Sexual Behavior, Risk Factors, Socioeconomic Factors

## Abstract

**OBJECTIVE:**

To estimate the prevalence of high-risk human papillomavirus (HR-HPV) anal infection and associated factors in human immunodeficiency virus (HIV) positive patients in Medellín.

**METHODS:**

Descriptive cross-sectional study in 300 HIV-positive patients, adults, with history of anal intercourse, treated in two health care services of Medellín 2017–2018. We conducted a structured survey on sociodemographics, sexual behavior and medical history. HPV was detected in anal swabs tested by the COBAS 4800 system. Exploratory data analysis of risk factors associated with HR-HPV was conducted by chi-square test of independence and both raw and adjusted prevalence ratios used the Poisson regression model, at a 95% confidence interval.

**RESULTS:**

The high-risk HPV had a prevalence of 82.7%; HPV16 had a prevalence of 32.7%, HPV18 a prevalence of 21.7% and other HPV types scored 78.3%. The high-risk HPV prevalence in women was of 68.2% and 83.8% in men. The risk factors associated with high-risk HPV after adjustment were age under 30 years, elementary education, casual sex partners, and first sexual activity before 18 years old.

**CONCLUSIONS:**

The high incidence of high-risk HPV, along with the occurrence of coinfections by multiple types in the study population shows their susceptibility to develop some type of anal intra-epithelial neoplasia. It is important to establish sexual health programs focused on primary health care.

## INTRODUCTION

Anal cancer is a matter of interest for public health, especially for the HIV-positive population, over whom the adjusted incidence per age is at 30 per 100,000 person-years in women and at 130 in men who have sex with men (MSM) ^1 , 2^ .

The etiology of anal cancer is associated with the persistent infection by the human papillomavirus (HPV), present in more than 80% of the cases. The most frequent virus is the 16 genotype, detected in nearly 70% of biopsies ^3^ . Anal intercourse and high number of sexual partners are important risk factors in the direct transmission of the virus, through the contact with the squamous epithelium and the anal transitional zone. Likewise, human immunodeficiency virus (HIV) concomitant infection and the use of highly active antiretroviral therapy (HAART) increase the survival rate of patients, promote HPV persistence and progress intraepithelial lesions. Thus, anal cancer is one of the most important non-AIDS-defining cancers ^1 , 5 , 6^ .

The prevalence of HPV infection in the anal canal is high for HIV-positive people, and even greater in men who have sex with men, where rates range between 40% and 92% ^7 , 8 , 9^ . Epidemiological, pathogenical and clinical characteristics of the anal cancer are similar to those of cervical cancer. This leads to the incorporation of several strategies for early detection of anal cancer: anal cytology, high resolution anoscopy (HRA) and biopsy ^10^ , which are contemplated by the anal cancer early detection program proposed in the United States for the high-risk population, HIV-positive people included ^11^ .

Colombia lacks structured protocols for the prevention of anal neoplasia, despite the increasing trend of HIV infection. According to the Colombian Ministry of Health, 46,348 new HIV cases were reported in 2013, rising up to 61,147 in 2015, which equals a 0.1% prevalence. These figures account for a population at risk of infection by HPV and premalignant lesions in the anal canal ^12 , 13^ .

The cases of anal cancer are reported within the colon and rectal cancer group in Colombia, according to the CIE 10-C21 classification. It hides the extent of the problem, as it prevents the availability of epidemiological information regarding the behavior of the anal neoplasia and HPV infection, important data to implement prevention and early-detection programs ^14^ . This study estimates the prevalence of high-risk HPV infection in the anal canal in HIV-positive patients and its relation with epidemiological, clinical and sexual behavior factors. These evidences can support the importance of establishing diagnosis and treatment protocols for anal intra-epithelial neoplasia (AIN) within this specific population.

## METHODS

Descriptive study conducted with 300 participants over 18 years old, HIV-positive and with a history of anal intercourse, selected out of 2,000 patients treated in two health care services offering HIV programs in Medellín. These services referred patients to the Cytology Service of the School of Microbiology from Universidad de Antioquia, where they were invited to join the research from June 2016 to December 2018.

The referred patients received information on the research. Each individual answered a structured questionnaire, previously validated in form and content and subjected to pilot testing. The questionnaire was divided into survey blocks: sociodemographic characteristics, risk factors, sexual behavior, signs and symptoms and information about the tests.

The evaluated factors were: sexual partner, being considered as regular sexual partner those with whom a stable affective relationship is maintained; and as casual sexual partner those with whom casual sex happens, without affection; in such case, there may be one or several partners. Tobacco or alcohol consumption: those who consume these substances or had done it in the past, regardless of frequency. Condom use: frequent or regular condom use. AIN symptoms: rectal pain, rectal bleeding, feeling of lumps in the rectum or abnormal rectum discharges the participant may had experienced at some point in life. History of benign lesion, and any anal or perianal lesion, such as anal fissures, fistulas, warts or abscesses.

An anal swab was collected from each participant, by a cervical brush, and the samples were deposited in microscope slides, according to the guidelines of Darragh T et al. ^15 , 16^ . The remaining material in the cervical brush was deposited in a vial filled with Thin Prep PreservCyt solution, orbitally shaken for several seconds for cells to be obtained. This sample was analyzed in the Infection and Cancer Group of Universidad de Antioquia using the COBAS 4800® system (Roche Diagnostics), that simultaneously identifies the types HPV16 and HPV18 along with the other 12 high-risk genotypes (31, 33, 35, 39, 45, 51, 52, 56, 58, 59, 66 and 68). This method amplifies a 200bp segment of the HPV L1 gene, identified by genotype-specific PCR fluorescent oligonucleotides probes in real time ^17^ . The results of the sample were: either positive or negative for HPV16, HPV18 or other high-risk genotypes. The results were informed to each participant and to the health care service providing treatment.

We realized a descriptive analysis with median and mode measures of central tendency, and inter-quartile range as measure of dispersion. We created three HPV groups, one for the 16 genotype, one for the 18 genotype and one for others HR-HPV, determining the frequencies according to the sociodemographic characteristics. We investigated the correlation, by comparing the risk factor occurrence to HPV infection, using the Chi-square test of independence, the Fisher’s exact test and Chi-square distribution. The raw prevalence ratios were calculated at a significance level of 0.05.We proposed three Poisson regression models, having the presence or absence of HR-HPV as the outcome variable, the first one including HPV16, the second including HR-HPV18 and the third one encompassing all high-risk genotypes. We included the factors at p < 0.25 in the model with bivariate analysis. The confidence intervals were set at 95% and the statistical significance p < 0.05, both for bivariable and multivariable determinants. The statistical analyses were performed in the Stata Release 15 and EpiData 3.1 version software.

This research was authorized by the Bioethics Committee of the Facultad Nacional de Salud Pública de la Universidad de Antioquia under an act dated July 9, 2015, which was updated by the act no. CI00328, dated August 9, 2018. According to the Resolution no. 8430 issued by the Colombian Ministry of Health, this research was classified as greater than minimal risk. The principles of data confidentiality, handling and custody were based on the Declaration of Helsinki and on The Council for International Organizations of Medical Sciences (CIOMS) guidelines. The participants signed off an informed consent form.

## RESULTS

Of the 300 participants, 92.7% were men. The average age was 39 years old for men and 45 years old for women. Among participants, 49.7% were aged between 30 and 50 years old. Ninety five percent lived in the urban area and 84.5% had a middle-low socioeconomic status, 56% attended college and 54% were covered by the contributory health care system ( Table 1 ).

**Table 1:** HR-HPV infection according to HIV-positive patients sociodemographic characteristics. Medellín, 2018.

Characteristics		VPH16		VPH18		Others HR-HPV		> 2HR-HPV		HR-HPV	
		n	%	n	%	n	%	n	%	n	%
Gender	Female (n = 22)	5	22.7	3	13.6	13	59.1	1	4.6	15	68.2
	Male (n = 278)	93	33.5	62	22.3	222	79.9	30	10.8	233	83.8
Age	< 30 years old (n = 73)	34	46.6	32	43.8	66	90.4	20	27.4	66	90.4
	30–50 years old (n = 149)	47	31.5	25	16.8	121	81.2	9	6.0	129	86.6
	> 50 years old (n = 78)	17	21.8	8	10.3	48	61.5	2	2.6	53	68.0
Place of residence	Urban (n = 285)	93	32.6	64	22.5	223	78.3	30	10.5	235	82.4
	Rural (n = 15)	5	33.3	1	6.7	12	80.0	1	6.7	13	86.7
Health insurance	Contributory (n = 162)	55	34.0	36	22.2	131	80.9	20	12.3	138	85.2
	Subsidized (n = 138)	43	31.1	29	21.0	104	75.4	11	8.0	110	79.7
Socioeconomic strata	1–3 Middle low (n = 251)	78	31.1	59	23.5	196	78.1	26	10.4	207	82.5
	4–6 Middle high (n = 46)	19	41.3	5	10.9	38	82.6	4	8.7	40	87.0
Educational level	College (n = 168)	66	39.3	41	24.4	144	85.7	24	14.3	150	89.3
	Secondary education (n = 90)	24	26.7	22	24.4	68	75.6	7	7.8	72	80.0
	Primary education	8	20.0	2	5.0	22	55.0	0	0.0	25	62.5
Total		98	32.7	65	21.7	235	78.3	31	10.3	248	82.7

**Graph 1:**
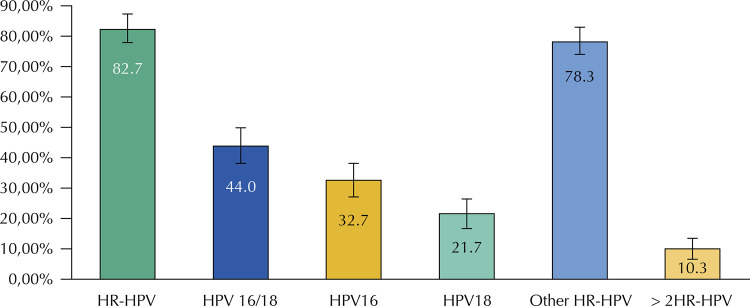
Anal infection caused by different types of oncogenic HPV prevalence in HIV-positive patients. Medellín, 2018.

HR-HPV: positive result for at least one of the 14 high risk genotypes analyzed. HPV16-18: positive result for either HPV16 or HPV18 or both; HPV16: positive result at least for HPV16; HPV18: positive result for at least HPV18; other HR-HPV: positive result for at least one of the following types: 31, 33, 35, 39, 45, 51, 52, 56, 58, 59, 66 y 68. > 2HR-HPV: positive result for HPV16, HPV18 and at least one of the others HR-HPV. The categories are not mutually exclusive, thus the total percentages add up to over 100.

The HR-HPV infection prevalence (of at least one of the detectable genotypes by the exam) was of 82.7%, 95%CI 78.2–87.1; VPH16 prevalence was of 32,7%, 95%CI 27.2–38.1; VPH18 prevalence was of 21.7%, 95%CI 16.8–26.5 and other genotypes prevalence was of 78.3%, 95%CI 73.5–83.2. The prevalence of HPV infection by multiple genotypes, three or more, was 10.3%, 95%CI 6.72-13.94 (Figure 1).

The bivariate analysis shows the prevalence of infection by the HPV types that the exam detects. The occurrence of HR-HPV, HPV16 and other types of HR-HPV was higher in men under 30 years old, insured by the contributory regime, middle-high stratum and who had completed higher education. The infection by HPV18 y > 2HPV was greater in the middle-low stratum. Despite the low number of participants residing in the rural area, the HPV genotypes distribution was similar in both places of residence, with the exception of HPV18, which was notoriously smaller in the rural area (6.7%) than the urban (22.5%) ( Table 1 ).

The average age of first sexual intercourse was 15 years old and 17 years old for the first anal intercourse, without significant difference per gender. The average time elapsed since HIV diagnosis was 6.9 years. Of the participants, 98.7% were receiving HAART therapy. Those with casual sexual partners presented higher risk of infection by HPV16. Those who had sex in exchange for money at some point, tobacco users and those who had not frequently used condoms presented a higher risk of HPV18 infection. The infection by HR-HPV was related to: casual sexual partners, regular sexual partners, first sex intercourse before 18 years old, history of 10 plus sexual partners and history of benign lesions, such as anal fissures, ulcers, abscesses and fistulas. Having a HIV diagnosis in the past three years or more was a protective factor against infection by HR-HPV, HPV16 and HPV18 ( Table 2 ).

**Table 2:** Prevalence of anal infection by HR-HPV related to sexual and medical factors in HIV-positive patients. Medellín, 2018.

Characteristics		HPV16 infection			HPV18 infection			HR-HPV infection		
		N	%	PR (95%CI)	n	%	PR (95%CI)	n	%	RP (CI95%)
Sexual partner	No sex partner (n = 98)	24	24.5	Ref.	20	20.4	Ref.	69	70.4	Ref.
	Regular (n = 116)	38	32.8	1.3 (0.9–2.07) ^c^	24	20.7	1.0 (0.6–1.7) ^c^	99	85.3	**1.2 (1.0–1.4)** ^b§^
	Casual (n = 86)	36	41.9	**1.7 (1.1–2.6)** ^a,c^	21	24.4	1.2 (0.7–2.1) ^c^	79	91.9	**1.3 (1.1–1.5)** ^b,c^
Age of first sexual intercourse	≥ 18 years (n = 77)	19	24.7	Ref.	14	18.2	Ref.	56	72.7	**Ref.**
	< 18 years (n = 223)	79	35.4	1.4 (0.9–2.2)	51	22.9	1.3 (0.7–2.1)	192	86.1	**1.2 (1.0–1.4)** ^b^
Sex in exchange for money	No (n = 238)	77	32.4	Ref.	40	16.8	**Ref.**	194	81.5	Ref.
	Yes (n = 61)	21	34.4	1.1 (0.7–1.6)	25	41.0	**2.4 (1.6–3.7)** ^b^	54	88.5	1.1 (0.9–1.2)
Tobacco	Non-tobacco user (n = 179)	59	33.0	Ref.	31	17.3	**Ref.**	144	80.4	Ref.
	Tobacco user (n = 121)	39	32.2	1.0 (0.7–1.4)	34	28.1	**1.6 (1.1–2.5)** ^a^	104	86.0	1.1 (0.9–1.2)
Alcoholic beverages	Non-drinker (n = 79)	23	29.1	Ref.	14	17.7	Ref.	61	77.2	Ref.
	Drinker (n = 221)	75	33.9	1.2 (0.8–1.7)	51	23.1	1.3 (0.8–2.2)	187	84.6	1.1 (0.9–1.3)
Psychoactive substances	Nonuser (n = 212)	66	31.1	Ref.	44	20.8	Ref.	173	81.6	Ref.
	User (n = 88)	32	36.4	1.2 (0.8–1.6)	21	23.9	1.2 (0.7–1.8)	75	85.2	1.0 (0.9–1.2)
Number of sexual partners	≤ 10 (n = 97)	29	29.9	Ref.	17	17.5	Ref.	73	75.3	**Ref.**
	> 10 (n = 202)	69	34.2	1.1 (0.8–1.6)	48	23.8	1.4 (0.8–2.2)	175	86.6	**1.2 (1.0–1.3)** ^a^
Condom use	Yes (n = 164)	48	29.3	Ref.	27	16.5	Ref.	136	82.9	Ref.
	No (n = 136)	50	36.8	1.3 (0.9–1.7)	38	27.9	**1.7 (1.1–2.6)** ^a^	112	82.4	1.0 (0.9–1.1)
STD history	No (n = 76)	24	31.6	Ref.	12	15.8	Ref.	58	76.3	Ref.
	Yes (n = 224)	74	33.0	1.1 (0.7–1.5)	53	23.7	1.5 (0.9–2.7)	190	84.8	1.1 (0.9–1.3)
Time elapsed since HIV diagnosis	< 3 years (n = 70)	30	42.9	**Ref.**	25	35.7	Ref.	65	92.9	**Ref.**
	≥ 3 years (n = 218)	64	29.4	**0.7 (0.5–0.9)** ^a^	37	17.0	**0.5 (0.3–0.7)** ^b^	172	78.9	**0.9 (0.8–0.9)** ^b,d^
AIN related symptoms	No (n = 141)	48	34.0	Ref.	27	19.1	Ref.	115	81.6	Ref.
	Yes (n = 159)	50	31.4	0.9 (0.7–1.3)	38	23.9	1.3 (0.8–1.9)	133	83.6	1.0 (0.9–1.1)
Benign lesions	No (n = 187)	54	28.9	Ref.	45	24.1	Ref.	148	79.1	Ref.
	Yes (n = 113)	44	38.9	1.4 (1.0–1.9)	20	17.7	0.7 (0.5–1.2)	100	88.5	**1.1 (1.0–1.2)** ^a^

PR: Prevalence ratio; HPV16: comprehends positive results for HPV16; HPV18: comprehends positive results for HPV18; Ref.: reference category.

^a^ p < 0.05

^b^ p < 0.01

^c^ Chi-square distribution

^d^ Fisher’s exact test

Note: In all analyses, except for the ordinal variables, the chi-square test of Independence was used.

The infection by HPV16 was related to age in the multivariate analysis using the Poisson regression model. The risk of infection reduced as the participants’ ages increased, especially over 50 years old, age in which the risk was lower than 50%; and the risk of infection was also related to having a casual sexual partner. The model was adjusted by gender. The infection by HPV18 was related to age, presenting the same pattern as HPV16, with the risk being lowered to 60% over 50 years old. The infection by HPV18 was also associated with sex in exchange for money, infrequent use of condoms and history of benign anal lesions. The infection by HR-HPV was connected to the educational level, the individuals with higher education and a sexual partner, whether regular or casual, presented a lower risk once this gender and age connection was adjusted ( Table 3 ).

**Table 3:** Factors associated with the oncogenic HPV infection in the anal canal of HIV-positive patients. Medellín, 018.

Variable	Category	HPV16			HPV18			HR-HPV		
		aPR	95%CI	p	aPR	95%CI	p	aPR	95CI%	p
Gender	Male	1.5	0.7–3.3	0.361	1.6	0.5–4.6	0.409	1.2	0.9–1.6	0.273
Female(Ref)										
Age	30–50 years old	0.7	0.5–1.0	**0.043**	0.5	0.3–0.7	**0.001**	1.0	0.9–1.1	0.319
< 30 years old (Ref)	> 50 years old	0.5	0.3–0.8	**0.006**	0.4	0.2–0.9	**0.001**	0.9	0.7–1.0	0.074
Educational level	Secondary education	-	-	-	-	-	-	0.9	0.8–1.1	0.314
Primary education (Ref)	Higher education	-	-	-	-	-	-	0.8	0.6–1.0	**0.044**
Sexual partner	Regular	1.2	0.8–1.9	0.429	-	-	-	1.1	1.0–1.3	0.049
No sex partner (Ref)	Casual	1.6	1.0–2.4	**0.041**	-	-	-	1.2	1.1–1.4	**0.003**
Sex in exchange for money	Yes	-	-	-	2.0	1.3–2.9	**0.001**	-	-	-
Condom use	No	-	-	-	1.6	1.0–2.4	**0.031**	-	-	-
Anal lesions	Yes	-	-	-	0.6	0.4–0.9	**0.019**	-	-	-

aPR: Adjusted prevalence ratio; Ref: reference category – PR value = 1. The statistically significant values are highlighted in bold. *Poisson regression model.

## DISCUSSION

The main sociodemographic characteristics of the participants are comparable with those of the HIV-positive patients at national level, which supports the representativeness of the sample, although it was not randomly selected. The majority of participants are insured by the contributory health care system, live in the urban area and over 75% are men ^18^ . The larger proportion of men in the study is due to the fact that they are, in large account, homosexuals, engaged in anal intercourse, from which they contracted HIV. In addition, in the male population anal intercourse is increasingly common, whether they are homo or bisexuals ^19^ .

The oncogenic HPV prevalence rate found in the 300 HIV-positive participants was 82.7%, which is higher than globally reported rates. Studies carried out in the Asian region report rates in HIV-positive men ranging between 45% and 74% in Taiwan ^20 , 21^ ; in Japan, a rate of 76% found in 421 MSM ^22^ ; in China, a rate of 61% found 212 MSM ^23^ and in Korea, a rate of 47% found in 201 men with the same characteristics ^24^ . In South Africa, Muller et al. ^25 , 26^ reported a prevalence of 58% found in 191 MSM, 81% found in HIV-positives and 39% in HIV-negative individuals, emphasizing the disparity in infection occurrence according to the serological status of participants. Studies show that this infection is 4 to 10 times more frequent in MSM than in heterosexual men, and the presence of HIV increases the risk.

The incidence of HR-HPV in the American continent also varies, as the Medina et al. ^28^ research in Puerto Rico shows, with 79% of prevalence in 239 HIV-positive participants and a low proportion of heterosexual men ^28^ . In the United States (USA), an incidence higher than 74% in MSM HIV-positive was found ^1 , 8 , 29^ . These rates are similar to those in this study. The high HR-HPV prevalence in MSM HIV-positive indicates a higher risk of anal cancer in this population group because the HIV infection increases their susceptibility to reinfections, persistence and infection reactivation by HPV ^5 , 30^ . Both are sexually transmitted diseases (STD), thus the associated risk behaviors for HIV can also influence the HPV infection ^2 , 23^ .

Studies focused on women are scarce, reporting variable infection rates. Of 138 Brazilian HIV-positive women, 43% tested positive for oncogenic HPV ^31^ , a rate lower than the one found in the USA (76%), where the rate almost doubled those of HPV cervical, both for HIV-positive and HIV–negative women ^8^ . In this study, the women proportion was low and, yet, the occurrence of HR-HPV in the anal canal was close to that reported in the USA. Also, the proportion of women who engaged in anal intercourse was similar in Brazil, USA and in this research. This emphasizes the need to assess other determinant aspects on anal infection by HPV in this population, such as history of gynecologic malignancies, an association supported by research conducted with women presenting uterinecervix neoplasias ^32^ .

The 16 and 18 HPV genotypes have a major involvement in the carcinogenesis, as they were detected in 85% and 7%, respectively, of invasive anal cancer biopsies. Their presences at this anatomical area are a risk factor for anal cancer in itself, as it increases when multiple genotypes are found ^3 , 4^ . The prevalence of 16 and 18HPV in this study was slightly higher than what was found in studies where variable prevalence’s are reported. In the USA, the infection rates by HR-HPV were of 47%, by HPV16 of 28%, by VPH18 were of 11%; in Italy, the infection rates by HPV16 were of 27% and by HPV18 of 14%; in Puerto Rico, where the same commercial exam for viral DNA detection we used was employed, a similar rate for other HPV types was found (79%), and rates were slightly lower for the 16 and 18 genotypes, with 28% and 16%, respectively ^28^ . The HPV18 rates found in this study are higher than what were found by other studies, showing possible greater importance of this genotype in our environment. The dissimilar results can show that HR-HPV distribution varies within the different MSM groups and according to the HIV status. Other explanation is the peculiar characteristics of each study population.

The diagnosis exams employed in the different analyzed studies were based on PCR, a high-sensitivity and specificity test for detection of the virus ^34^ , but the commercial kits used vary on their ability to identify the viral genotypes. This can lead to minimal differences in prevalence, especially regarding multiple types infections ^28 , 29^ . The COBAS 4800 method used in this study can only specifically detect HPV16 and 18, but is unable to determine possible multiple infections of the 12 other genotypes.

Likewise, distinctive characteristics of the study population and of the study itself can influence the findings. Studies with samplings smaller than 200 individuals reported low prevalence of HR-HPV ^23 , 24^ . In contrast, studies that included HIV population only, men or MSM only, regardless of their serological status, found high prevalence. Same results were reported by studies carried out in urology clinics, HIV clinics, etc ^25^ . These aspects have a decisive effect on the prevalence in groups with specific characteristics and also explains the high infection rates in this study, which included participants presenting the two major risk factors in the etiology of HPV infection: the immunodeficiency caused by HIV and anal intercourse.

Regarding the HR-HPV risk factors for HIV-positive patients explored in this study, the higher risk was found in individuals with casual sexual partners and first sexual intercourse before 18 years old. These factors are different from those found in other studies, such as the one conducted by Chia-Chun Lin, et al. in Taiwan ^35^ , where the main risk factor was history of STD, use of hallucinogens and presence of warts. Glick et al. ^36^ reported results similar to those of this study regarding the change of partners, by finding that, for each additional partner in the past three months, the infection prevalence increased 24% and the incidence, 32%. Also, Natera et al. ^37^ observed the risk increases if an individual has more than two sexual partners.

The higher prevalence of HR-HPV found in this study differs from other studies, probably due to the differences in the characteristics of the study population. In Taiwan, 51.4% were receiving HAART therapy and 33.3% engaged in anal intercourse ^21^ ; in Korea, 34% were women, of which 47.3% were 50 years old or older, an age where HPV infection significantly decreases; besides, 70% had not attended college, and proportion of homo/bisexual men was of 66.2% ^23^ . In the study conducted with Brazilian women, 52.2% of participants had anal intercourse once in their lifes ^31^ , while in this study, all participants were compliant with the criteria. In this same sense, in this study, the men proportion was high, the majority was receiving HAART therapy, all were HIV-positive and had anal intercourse as inclusion criteria.

It was difficult to compare the HPV infection incidence found with other studies, probably due to the sociodemographic and clinical differences among the participants, along with the inclusion criteria in each study. Yet, we were able to observe that age, educational level, anal receptive intercourse, casual sex partners and first sexual activity under the age of 18 are predictive factors for risk of HR-HPV infection. However, the time elapsed since the HIV diagnosis ^33^ worked as a protective factor, and the HAART therapy ^22^ was not significant in the statistical model we used. Thus, these variables must be analyzed in detail by further studies, because of their implication to the progression of AIN.

Using exams that analyze other HR-HPV genotypes altogether hinders an individual analysis. Thus, we could not extract the incidence data for each one of them in the study population, which would make the study richer.

Being a cross-sectional study, we were able to explore the possible risk factors implicated in the anal infection by oncogenic HPV. However, analytical epidemiologic studies are required to deepen the subject and detect the critical aspects to which prevention campaigns must aim. The viral detection method used did not identify the other 12 types of oncogenic HPV. Thus, the coinfections prevalence may be higher than reported because of the possible presence of more than one HPV strain in the participants. While the HIV infection status (CD4+ cell count and viral load) are greatly important aspects for HPV infection, satisfactory up-to-date data were not available to be included in the analysis.

The findings of this research allow a baseline of the HR-HPV infection in the anal canal of the HIV-positive population, which was unknown up to now in the Colombian context. The high prevalence of HR-HPV infection and, especially of the 16 and 18 types of evidence the strong exposure to these virus genotypes to which the HIV-positive population engaged in anal intercourse is subjected to. Consequently, the findings point to the risk of anal neoplasia and its possible progression to anal cancer; especially considering the multiple genotypes infections, which suggest their persistence over time, increasing the risk ^22^ . The strong presence of other oncogenic virus types, such as 31, 33, 35, 39, 45, 51, 52, 56, 58, 59, 66 and 68 in the anal canal of these patients shows the need to further explore the role they play in carcinogenesis. Such aspects must be considered in primary prevention strategies currently considered towards this population.
