# Supplementary material for: High-risk human papillomavirus infection and associated factors in the anal canal of HIV-positive patients in Medellín, 2017–2018
Source: Rev Saude Publica. 2020 Oct 23;54:93. doi: 10.11606/s1518-8787.2020054001692 (PMC7584410; doi:10.11606/s1518-8787.2020054001692)
Supplement: Translation

# Translation: Infección por el virus del papiloma humano de alto riesgo y factores asociados en canal anal de pacientes con VIH en Medellín, 2017–2018

**Authors:** Daniela Herrera Posada, Lucia Stella Tamayo Acevedo, Marleny Valencia Arredondo, Gloria Inéz Sánchez Vásquez

**Affiliations:** I Colombia Universidad de Antioquia. Escuela de Microbiología. Medellín, Antioquia, Colombia; II Colombia Universidad de Antioquia. Facultad de Medicina. Medellín, Antioquia, Colombia

**Keywords:** Infecciones por Paillomavirus, epidemiología, Infecciones por VIH, Neoplasia anal, Conducta Sexual, Factores de Riesgo, Factores Socioeconómicos

## Abstract

**OBJETIVO:**

Estimar la prevalencia de la infección anal por el virus del papiloma humano de alto riesgo y factores asociados en pacientes con el virus de inmunodeficiencia humana (VIH) de Medellín.

**MÉTODOS:**

Estudio descriptivo transversal en 300 pacientes VIH positivos, adultos, con historia de relaciones sexuales anales, atendidos en dos instituciones de salud de Medellín 2017-2018. Se aplicó una encuesta estructurada sobre características socio-demográficas, comportamiento sexual y antecedentes clínicos. El VPH se detectó en muestras de exfoliado anal mediante la prueba COBAS 4800. Se realizó análisis exploratorio de factores de riesgo asociados al VPH-AR mediante la prueba Chi cuadrado de independencia y razones de prevalencia cruda y ajustadas por regresión de Poisson, con intervalos del 95%.de confianza.

**RESULTADOS:**

La prevalencia global de VPHAR fue 82,7%; VPH 16 de 32,7%, VPH 18 de 21,7% y otros tipos 78,3%. La prevalencia de VPHAR en mujeres fue de 68,2% y en hombres, 83,8%. Los factores de riesgo asociados al VPH-AR luego del ajuste fueron tener menos de 30 años, algún grado de educación básica primaria, pareja ocasional e inicio de relaciones sexuales antes de 18 años.

**CONCLUSIONES:**

La alta frecuencia de infección por VPHAR, así como la existencia de co-infecciones por múltiples tipos en la población de estudio muestra la susceptibilidad que tienen para desarrollar algún grado de Neoplasia Intraepitelial Anal. Es importante establecer programas de promoción de la salud sexual con enfoque de atención primaria.

## INTRODUCCIÓN

El cáncer anal es un tema de interés para la salud pública, en particular en la población VIH positiva, en quienes se reporta incidencias ajustadas por edad de 30 por 100.000 años-persona en mujeres y de 130 en hombres que tienen sexo con hombres (HSH) ^1 , 2^ .

La etiología del cáncer anal se asocia a la infección persistente por el virus del papiloma humano (VPH), presente en más del 80,0% de los casos. Es más frecuente el genotipo 16, detectado aproximadamente en el 70,0% de las biopsias ^3^ . Las relaciones sexuales anales y el alto recambio de parejas sexuales son factores de riesgo importantes para la transmisión directa del virus al entrar en contacto con el epitelio escamoso y zona de transición anal. Asimismo, la infección concomitante por el virus de inmunodeficiencia humana (VIH) y el uso de terapia antirretroviral altamente efectiva (HAART) aumentan la tasa de sobrevivencia de los pacientes, favorece la persistencia del VPH, la instauración y progresión de las lesiones intraepiteliales. Por esta razón, el cáncer anal es uno de los cánceres no definitorios de SIDA más importante ^1 , 5 , 6^ .

La prevalencia de la infección por VPH en canal anal es alta en personas VIH positivas; y aún mayor en los hombres que tienen sexo con hombres, donde las cifras oscilan entre el 40,0% y 92,0% ^7 , 8 , 9^ . Características epidemiológicas, patogénicas y clínicas para el cáncer anal son similares a las de cáncer del cuello uterino. Eso lleva a la incorporación de estrategias de detección precoz para el cáncer anal: citología anal, anoscopia de alta resolución (AAR) y biopsia ^10^ , las cuales se encuentran contempladas en el programa de detección precoz para cáncer anal propuesto en Estados Unidos, a poblaciones de alto riego, entre ellas, personas VIH positivas ^11^ .

Colombia no cuenta con protocolos estructurados para la prevención de la neoplasia anal a pesar de que la infección por VIH tiende a crecer. Según estadísticas del Ministerio de Salud de Colombia, se reportaron 46.348 casos nuevos de VIH en el 2013, aumentando a 61.147 en el 2015, que corresponde a una prevalencia de 0,1%. Estas cifras dan cuenta de una población en riesgo de infectarse por VPH y de desarrollar lesiones premalignas en canal anal ^12 , 13^ .

Los casos de cáncer anal se reportan en el grupo de cáncer de colon y recto en Colombia, según la clasificación CIE 10-C21. Eso hace difícil visibilizar la magnitud del problema pues impide disponer de información epidemiológica en lo referente al comportamiento de la neoplasia anal y la infección por VPH, datos importantes al momento de implementar programas de prevención y detección precoz ^14^ . Esta investigación tuvo como objetivo estimar la prevalencia de la infección por VPH de alto riesgo oncogénico en canal anal de pacientes VIH positivos y la relación con factores epidemiológicos, clínicos y de comportamiento sexual. Esas evidencias poden sustentar la importancia de establecer protocolos de diagnóstico y de tratamiento de Neoplasia Intraepitelial Anal (NIA) en esta población en específico.

## MÉTODOS

Estudio descriptivo, realizado en 300 participantes mayores de 18 años con diagnóstico previo de VIH e historia de relaciones sexuales anales, seleccionados de una población de 2.000 pacientes atendidos en dos instituciones de salud que cuentan con programas de VIH en Medellín. Estas instituciones remitieron a los pacientes al Servicio de Citología de la Escuela de Microbiología de la Universidad de Antioquia, en donde fueron invitados a participar en la investigación de junio de 2016 a diciembre de 2018.

Los pacientes remitidos fueron sensibilizados con información acerca de la investigación. Cada individuo respondió una encuesta estructurada dirigida, previamente validada por apariencia y contenido y sometida a prueba piloto. La encuesta fue dividida por los bloques de preguntas: características sociodemográficas, factores de riesgo, conductas sexuales, signos y síntomas y conocimientos sobre la citología.

Los factores evaluados fueron: pareja sexual, se tomó como pareja permanente a aquella con la que se tiene una relación estable mediada por el afecto, y como pareja ocasional, con la/s que se tiene relaciones sexuales eventuales sin involucrar sentimientos afectivos; en tal caso puede ser una o varias parejas ocasionales. Exposición a tabaco o bebidas alcohólicas: quienes consumen estas sustancias o lo hicieron en el pasado, independientemente de la frecuencia del consumo. Uso del condón: uso frecuente o de manera regular del mismo. Los síntomas relacionados con NIA: dolor rectal, sangrado rectal, sensación de masa en el ano o secreciones anormales en la zona, que el participante haya podido tener en algún momento de su vida. Antecedente de lesiones benignas, a toda lesión en la región anal o perianal como fisuras, fístulas, condilomas o abscesos.

A cada participante se le tomó muestra de exfoliado anal utilizando citocepillo ginecológico, la muestra se extendió en láminas de vidrio para citología, siguiendo las recomendaciones de Darrag T et al ^15 , 16^ . El remanente del citocepillo se depositó en un vial con solución de ThinPrep PreservCyt, agitándose circularmente durante varios segundos para la obtención de células. Esta muestra fue evaluada en el laboratorio del Grupo Infección y Cáncer de la Universidad de Antioquia por la prueba COBAS® 4800 (Roche Diagnostics), la cual identifica en forma simultánea los genotipos VPH16 y VPH18 y en conjunto los otros 12 genotipos de alto riesgo (31, 33, 35, 39, 45, 51, 52, 56, 58, 59, 66 y 68). Este método amplifica un segmento de 200pb del gen L1 del VPH, identificado con sondas de oligonucleótidos fluorescentes específicas de genotipo en PCR en tiempo real ^17^ . El resultado de la prueba se reportó: positivo o negativo para VPH16, VPH18 y otros genotipos de alto riesgo. Los resultados del examen fueron informados a cada participante y a la institución tratante.

Se realizó análisis descriptivo mediante medidas de tendencia central como mediana y moda, de dispersión como rango intercuartílico. Se conformaron tres grupos de VPH, uno para el genotipo 16, otro para el genotipo 18 y el otro para el resto de VPH-AR, determinando las frecuencias según las características socio-demográficas. Se exploró la relación, a través de la comparación de la frecuencia de los factores de riesgo con la infección por VPH, mediante la prueba Chi cuadrado de independencia, exacta de Fisher y la prueba Chi cuadrado de tendencia lineal. Se calcularon razones de prevalencia cruda con un nivel de significación de 0,05. Se plantearon tres modelos de regresión Poisson, tomando como variable de desenlace la presencia o ausencia de VPH-AR, el primero incluyó VPH16, el segundo VPH18 y el tercero todos los genotipos de alto riesgo. Se incluyeron los factores con p < 0,25 en los modelos en el análisis bivariado. Los intervalos de confianza se establecieron en el 95% y la significación estadística en el p <0,05, tanto para determinaciones bivariadas como multivariadas. El análisis estadístico se realizó en los programas Stata, Versión 15 y EPidat versión 3.1.

Esta investigación contó con el aval del Comité de Bioética de la Facultad Nacional de Salud Pública de la Universidad de Antioquia en el acta del 9 de julio de 2015 y actualizado en el acta CI00328 del 9 de agosto del 2018. Según la Resolución 8430 del Ministerio de Salud de Colombia, la investigación se catalogó como de riesgo mayor al mínimo. Los principios de confidencialidad, manejo y custodia de la información fueron basados en la declaración de Helsinki y las normas de The Council for International Organizations of Medical Sciences (CIOMS). Los participantes firmaran el termo de consentimiento informado.

## RESULTADOS

El 92,7% de los 300 participantes eran hombres. La mediana de edad fue 39 años para hombres y 45 años para mujeres. El 49,7% tenían entre 30 y 50 años. El 95,0% residían en zona urbana, el 84,5% pertenecían al nivel socioeconómico medio-bajo y el 56,0% habían accedido a educación superior; el 54,0% pertenecían al régimen contributivo de salud ( Tabla 1 ).

**Tabla 1:** Infección por VPH-AR según características socio-demográficas de pacientes VIH positivos. Medellín, 2018.

Características		VPH16		VPH18		Otros VPH-AR		> 2VPH-AR		VPH-AR	
		n	%	n	%	n	%	n	%	n	%
Sexo	Mujer (n = 22)	5	22,7	3	13,6	13	59,1	1	4,6	15	68,2
	Hombre (n = 278)	93	33,5	62	22,3	222	79,9	30	10,8	233	83,8
Edad	< 30 años (n = 73)	34	46,6	32	43,8	66	90,4	20	27,4	66	90,4
	30–50 años (n = 149)	47	31,5	25	16,8	121	81,2	9	6,0	129	86,6
	> 50 años (n = 78)	17	21,8	8	10,3	48	61,5	2	2,6	53	68,0
Residencia	Urbano (n = 285)	93	32,6	64	22,5	223	78,3	30	10,5	235	82,4
	Rural (n = 15)	5	33,3	1	6,7	12	80,0	1	6,7	13	86,7
Régimen de seguridad	Contributivo (n = 162)	55	34,0	36	22,2	131	80,9	20	12,3	138	85,2
	Subsidiado (n = 138)	43	31,1	29	21,0	104	75,4	11	8,0	110	79,7
Estrato socio-económico	1–3 Medio bajo (n = 251)	78	31,1	59	23,5	196	78,1	26	10,4	207	82,5
	4–6 Medio alto (n = 46)	19	41,3	5	10,9	38	82,6	4	8,7	40	87,0
Nivel educativo	Educación superior (n = 168)	66	39,3	41	24,4	144	85,7	24	14,3	150	89,3
	Básica secundaria (n = 90)	24	26,7	22	24,4	68	75,6	7	7,8	72	80,0
	Básica primaria (n = 40)	8	20,0	2	5,0	22	55,0	0	0,0	25	62,5
Total		98	32,7	65	21,7	235	78,3	31	10,3	248	82,7

La prevalencia global de infección por VPH-AR (al menos uno de los genotipos que detecta la prueba) fue 82,7%, IC95% 78,2–87,1, VPH16 de 32,7%, IC95% 27,2–38,1, VPH18 de 21,7%, IC95% 16,8–26,5 y para otros genotipos 78,3%, IC95% 73,5–83,2. La prevalencia de infección por VPH con múltiples genotipos, tres o más, fue de 10,3%, IC95% 6,72–13,94 (Figura 1).

El análisis bivariado muestra la prevalencia de infección por los tipos de VPH que detecta la prueba. La frecuencia de VPH-AR, VPH16 y otros tipos de VPH-AR fue mayor en hombres menores de 30 años, del régimen contributivo, estrato medio-alto y con educación superior. La infección por VPH18 y > 2VPH se encontró en mayor proporción en estrato medio bajo. A pesar del bajo número de participantes residentes en el área rural, la distribución de los genotipos de VPH fue similar en ambas zonas de residencia, con excepción del VPH 18 que fue notoriamente menor en la zona rural (6,7%) respecto a la urbana (22,5%) ( Tabla 1 ).

**Gráfico:** Prevalencia global de infección anal por diferentes tipos de VPH oncogénico en pacientes VIH positivos. Medellín, 2018.

VPH-AR: prueba positiva para al menos uno de los 14 genotipos de alto riesgo analizados. VPH16/18: prueba positiva para VPH16 o VPH18 o ambos; VPH 16: prueba positiva al menos para VPH 16, VPH 18: prueba positiva al menos para VPH 18, Otros VPH-AR: prueba positiva para al menos uno de los siguientes tipos: 31, 33, 35, 39, 45, 51, 52, 56, 58, 59, 66 y 68. > 2VPH-AR: prueba positiva para VPH16, VPH18 y al menos uno de los otros VP-AR. Las categorías no son excluyentes, por tanto la suma de los porcentajes no da 100.

La mediana de edad de inicio de relaciones sexuales fue 15 años y para el inicio de relaciones anales de 17 años, sin diferencias estadísticas significativas por sexo. La mediana del tiempo del diagnóstico de VIH fue 6,9 años. El 98,7% de los participantes recibían HAART. El mayor riesgo de infección por VPH16 ocurrió en personas con pareja ocasional. La infección por VPH18 se presentó con mayor riesgo entre personas que tuvieron sexo por dinero alguna vez en su vida, consumidores de tabaco y quienes no usaban el condón permanentemente en sus relaciones sexuales. La infección por VPH-AR se relacionó con: tener pareja sexual ocasional, pareja permanente, inicio de relaciones sexuales antes de 18 años, historia de más de 10 parejas sexuales y con antecedentes de lesiones benignas como fisuras, ulceras, abscesos y fístulas en el ano. Tener un diagnóstico de VIH hace tres años o más, fue factor protector tanto para la infección por VPH-AR, como para VPH 16 y 18 ( Tabla 2 ).

**Tabla 2:** Prevalencia de infección anal por VPH-AR relacionada con factores sexuales y clínicos en los pacientes VPH positivos, Medellín, 2018.

Característica		Infección por VPH16			Infección por VPH18			Infección por VPH-AR		
		n	%	RP (IC95%)	n	%	RP (IC95%)	n	%	RP (IC95%)
Pareja sexual	Sin pareja (n = 98)	24	24,5	Ref.	20	20,4	Ref.	69	70,4	Ref.
	Permanente (n = 116)	38	32,8	1,3 (0,9–2,07) ^c^	24	20,7	1,0 (0,6–1,7) ^c^	99	85,3	**1,2 (1,0–1,4)** ^b§^
	Ocasional (n = 86)	36	41,9	**1,7 (1,1–2,6)** ^a,c^	21	24,4	1,2 (0,7–2,1) ^c^	79	91,9	**1,3 (1,1–1,5)** ^b,c^
Edad inicio relaciones sexuales	≥ 18 años (n = 77)	19	24,7	Ref.	14	18,2	Ref.	56	72,7	**Ref.**
	< 18 años (n = 223)	79	35,4	1,4 (0,9–2,2)	51	22,9	1,3 (0,7–2,1)	192	86,1	**1,2 (1,0–1,4)** ^b^
Sexo por dinero	No (n = 238)	77	32,4	Ref.	40	16,8	**Ref.**	194	81,5	Ref.
	Sí (n = 61)	21	34,4	1,1 (0,7–1,6)	25	41,0	**2,4 (1,6–3,7)** ^b^	54	88,5	1,1 (0,9–1,2)
Tabaco	No consumo (n = 179)	59	33,0	Ref.	31	17,3	**Ref.**	144	80,4	Ref.
	Consumo (n = 121)	39	32,2	1,0 (0,7–1,4)	34	28,1	**1,6 (1,1–2,5)** ^a^	104	86,0	1,1 (0,9–1,2)
Bebidas alcohólicas	No consumo (n = 79)	23	29,1	Ref.	14	17,7	Ref.	61	77,2	Ref.
	Consumo (n = 221)	75	33,9	1,2 (0,8–1,7)	51	23,1	1,3 (0,8–2,2)	187	84,6	1,1 (0,9–1,3)
Sustancias psicoactivas	No consumo (n = 212)	66	31,1	Ref.	44	20,8	Ref.	173	81,6	Ref.
	Consumo (n = 88)	32	36,4	1,2 (0,8–1,6)	21	23,9	1,2 (0,7–1,8)	75	85,2	1,0 (0,9–1,2)
Número parejas sexuales	≤ 10 (n = 97)	29	29,9	Ref.	17	17,5	Ref.	73	75,3	**Ref.**
	> 10 (n = 202)	69	34,2	1,1 (0,8–1,6)	48	23,8	1,4 (0,8–2,2)	175	86,6	**1,2 (1,0–1,3)** ^a^
Uso del condón	Sí (n = 164)	48	29,3	Ref.	27	16,5	Ref.	136	82,9	Ref.
	No (n = 136)	50	36,8	1,3 (0,9–1,7)	38	27,9	**1,7 (1,1–2,6)** ^a^	112	82,4	1,0 (0,9–1,1)
Antecedente de ITS	No (n = 76)	24	31,6	Ref.	12	15,8	Ref.	58	76,3	Ref.
	SÍ (n = 224)	74	33,0	1,1 (0,7–1,5)	53	23,7	1,5 (0,9–2,7)	190	84,8	1,1 (0,9–1,3)
Tiempo con diagnóstico de VIH	< 3años (n = 70)	30	42,9	**Ref.**	25	35,7	Ref.	65	92.9	**Ref.**
	≥ 3años (n = 218)	64	29,4	**0,7 (0,5–0,9)** ^a^	37	17,0	**0,5 (0,3–0,7)** ^b^	172	78.9	**0,9 (0,8–0,9)** ^b,d^
Síntomas relacionados con NIA	No (n = 141)	48	34,0	Ref.	27	19,1	Ref.	115	81,6	Ref.
	Sí (n = 159)	50	31,4	0,9 (0,7–1,3)	38	23,9	1,3 (0,8–1,9)	133	83,6	1,0 (0,9–1,1)
Lesiones benignas	No (n = 187)	54	28,9	Ref.	45	24,1	Ref.	148	79,1	Ref.
	Sí (n = 113)	44	38,9	1,4 (1,0–1,9)	20	17,7	0,7 (0,5–1,2)	100	88,5	**1,1 (1,0–1,2)** ^a^

RP: razón de prevalencias; VPH16: incluye a las pruebas positivas para VPH16; VPH18: incluye a las pruebas positivas para VPH18; Ref.: categoría de referencia para el análisis.

^a^ p < 0,05

^b^ p < 0,01

^c^ Prueba de tendencia lineal

^d^ Prueba exacta de Fisher

Nota: Para todos los análisis, a excepción de las variables ordinales, se empleó la prueba Chi2 de independencia.

La infección por VPH16 estuvo en función de la edad en el análisis multivariado por regresión de Poisson. El riesgo de la infección fue menor a medida que aumentó la edad de los participantes, especialmente después de los 50 años, edad en la cual el riesgo fue menor del 50%; también estuvo en función de tener pareja sexual ocasional. El modelo fue ajustado por sexo. La infección por VPH18 estuvo en función de la edad, con el mismo patrón que VPH16, disminuyendo el riesgo en un 60%, después de los 50 años. La infección por VPH18 se asoció también con tener sexo por dinero, no usar condón de forma permanente y tener antecedentes de lesiones anales benignas. La infección por un VPH-AR estuvo en función del nivel educativo, siendo menor el riesgo en personas con educación superior y tener pareja sexual ya sea permanente u ocasional; una vez ajustada esta relación por sexo y edad ( Tabla 3 ).

**Tabla 3:** Factores asociados* a la infección por VPH oncogénico en canal anal de pacientes VIH positivos, Medellín, 2018.

Variable	Categoría	VPH16			VPH18			VPH-AR		
		RPa	IC95%	p	RPa	IC95%	p	RPa	IC95%	p
Sexo	Hombre	1,5	0,7–3,3	0,361	1,6	0,5–4,6	0,409	1,2	0,9–1,6	0,273
Mujer (Ref)										
Edad	30–50 años	0,7	0,5–1,0	**0,043**	0,5	0,3–0,7	0,001	1,0	0,9–1,1	0.319
< 30 años (Ref)	> 50 años	0,5	0,3–0,8	**0,006**	0,4	0,2–0,9	0,001	0,9	0,7–1,0	0,074
Nivel educativo	Básica secundaria	-	-	-	-	-	-	0,9	0,8–1,1	0,314
Básica primaria (Ref)	Educación superior	-	-	-	-	-	-	0,8	0,6–1,0	**0,044**
Pareja sexual	Permanente	1,2	0,8–1,9	0,429	-	-	-	1,1	1,0–1,3	0,049
Sin pareja (Ref)	Ocasional	1,6	1,0–2,4	**0,041**	-	-	-	1,2	1,1–1,4	**0,003**
Sexo por dinero	Sí	-	-	-	2,0	1,3–2,9	**0,001**	-	-	-
Uso condón	No	-	-	-	1,6	1,0–2,4	**0,031**	-	-	-
Lesiones ano	Sí	-	-	-	0,6	0,4–0,9	**0,019**	-	-	-

RPa: razón de prevalencia ajustada; Ref: categoria de referencia - valor RP = 1.

Los valores con significación estadística se muestran en negrita

* Regresión de Poisson.

## DISCUSIÓN

Las principales características sociodemográficas de los participantes son comparables a las de los pacientes con VIH a nivel nacional; esto respalda la representatividad de la muestra, a pesar de no haber sido seleccionada aleatoriamente. La mayoría pertenece al régimen contributivo de salud, reside en zona urbana y más del 75,0% son hombres ^18^ . Asimismo, la mayor proporción de hombres en el estudio se puede explicar por el hecho de que fueron pacientes en un alto porcentaje homosexuales, con práctica de relaciones anales, mecanismos por el cual adquirieron la infección por VIH. Además, porque en la población masculina son cada vez más comunes las relaciones anales ya sean homo/bisexuales ^19^ .

La prevalencia de VPH oncogénico hallada en los 300 participantes VIH positivos fue del 82,7%, superior a las reportadas en el ámbito mundial. Los estudios realizados en la Región Asiática presentan cifras en hombres con VIH que oscilan entre 45,0 y 74,0% en Taiwán ^20 , 21^ , en Japón, prevalencias del 76,0% en 421 HSH ^22^ , en China del 61,0% en 212 HSH ^23^ y en Corea de 47,0% en 201 hombres con las mismas características ^24^ . En Sur África, Muller et al ^25 , 26^ reportan una prevalencia de 58,0% en 191 HSH, 81,0% en VIH positivos y 39,0% en VIH negativos, destacando la diferencia en la frecuencia de la infección según el estado serológico de los participantes. Estudios muestran que esta infección es entre cuatro y 10 veces más frecuente en HSH que en los hombres heterosexuales, riesgo que se incrementa con la condición de VIH ^27^ .

La frecuencia de VPH-AR en el continente americano también es variable, como lo muestra el estudio de Medina et al ^28^ en Puerto Rico, con un 79,0% en 239 participantes con VIH y una proporción de hombres heterosexuales baja ^28^ . En Estados Unidos (EU), se encontró una frecuencia superior al 74,0% en HSH, VIH positivos ^1 , 8 , 29^ . Estas cifras son similares a las del presente estudio. La alta prevalencia de VPH AR en los HSH-HIV positivos indican un riesgo mayor de desarrollar cáncer anal de este grupo poblacional, debido a que la infección por VIH incrementa la susceptibilidad para reinfecciones, persistencia y reactivación de infección por VPH ^5 , 30^ . Ambas son infecciones de transmisión sexual (ITS), así las conductas asociadas al riesgo de adquirir VIH también pueden influir en la infección por VPH ^2 , 23^ .

Son escasos los estudios con la población femenina, los cuales reportan frecuencias de infección variables. En 138 mujeres brasileñas VIH positivas, el 43,0% fue positiva para VPH oncogénicos ^31^ , cifra menor a la hallada en EU (76,0%), donde este valor casi duplicaba a la del VPH en el cérvix, tanto de mujeres positivas como negativas para VIH ^8^ . En la presente investigación, la proporción de mujeres fue baja y, sin embargo, la frecuencia de VPH-AR en el canal anal fue similar a la reportada en EU. Asimismo, la proporción de mujeres que practicaban sexo anal fue similar en Brasil, EU y en éste. Eso resalta la necesidad de evaluar en esta población otros aspectos determinantes para la infección anal por VPH, como tener antecedentes de neoplasias ginecológicas, cuya asociación ha sido sustentada en investigaciones realizadas con mujeres con neoplasias cervicouterinas ^32^ .

Los genotipos 16 y 18 del VPH tienen gran implicación en la génesis del cáncer, pues éstos se han detectado en biopsias de cáncer anal invasor en un 85,0% y 7,0% respectivamente. Su presencia en este sitio anatómico, por sí mismo, se constituye en un factor de riesgo para el desarrollo cáncer anal, el cual se incrementa cuando se encuentran múltiples genotipos ^3 , 4^ . La prevalencia de VPH16 y 18 en este estudio fue ligeramente superior a la observada en otras investigaciones donde se reportan prevalencias variables. La infección por múltiples VPH-AR fue de 47,0%, VPH16, 28,0% y VPH18, 11,0% ^29^ en EU; se encontró VPH16 en 27,0% y VPH18 en 14,0% en Italia ^33^ ; en Puerto Rico, donde emplearon la misma prueba comercial para la detección del ADN viral que se utilizó en la presente investigación, reportaron una frecuencia similar para otros tipos de VPH (79,0%), y levemente inferior para los genotipos 16 y 18 con 28,0% con 16,0%, respectivamente ^28^ . Las cifras de VPH 18 halladas en el presente estudio son superiores a las de las investigaciones analizadas, mostrando una posible importancia mayor de este genotipo en nuestro medio. Los resultados disímiles pueden indicar que la distribución de los VPH-AR varía en los distintos grupos de HSH y de acuerdo a su estado de HIV. Otra explicación, son las características propias de cada población evaluada.

Las pruebas de diagnóstico empleadas en los diferentes estudios analizados se fundamentan en la PCR, prueba con alta sensibilidad y especificidad para la detección del virus ^34^ , pero los kits comerciales utilizados varían en su capacidad para discriminar los genotipos virales. Esto puede dar lugar a diferencias mínimas en la prevalencia, especialmente para las infecciones por múltiples tipos ^28 , 29^ . El método COBAS 4800 del presente estudio solo detecta de forma específica el VPH 16 y 18, pero no permite determinar posibles infecciones múltiples entre los 12 genotipos restantes,

De igual manera, características propias de la población de estudio y de la investigación en sí pueden influir en las cifras halladas. Estudios con una muestra menor de 200 individuos reportaron prevalencias bajas de VPH-AR ^23 , 24^ . Por el contrario, aquellos estudios que incluyeron únicamente población VIH, solo hombres o HSH, independientemente de su estado serológico, hallaron altas prevalencias. Iguales resultados reportaron aquellas investigaciones realizadas en clínicas urológicas, clínicas de VIH, etc ^25^ . Estos aspectos tienen un efecto decisivo en la prevalencia en los grupos con características específicas y también explica el porqué de las altas cifras de infección en el presente estudio, donde se incluyeron participantes que cumplían con los dos factores de riesgo de mayor peso en la etiología de la infección por VPH: la inmunodeficiencia dada por el VIH y el sexo anal.

En cuanto a los factores de riesgo explorados para la infección por VPH-AR en pacientes con HIV del presente estudio, el mayor riesgo fue en personas con pareja ocasional e inicio de relaciones sexuales antes de los 18 años. Esos factores son diferentes a los encontrados por otros estudios, como el realizado por Chia-Chun Lin, et al en Taiwán ^35^ , donde el principal factor de riesgo fue tener historia de ITS, uso de drogas alucinógenas y presencia de verrugas. Glick et al ^36^ reportaron resultados similares al de la presente investigación con respecto al recambio de pareja, al encontrar que por cada compañero adicional en los últimos tres meses, la prevalencia de la infección aumentó en un 24,0% y la incidencia en un 32,0%. Asimismo, Natera et al ^37^ observaron que el riesgo aumenta si se tiene más de dos compañeros sexuales.

La mayor prevalencia de VPH-AR hallada en este estudio difiere de otras investigaciones, probablemente debido a las diferencias en las características de la población objeto. En Taiwán, el 51,4% recibía HAART y el 33,3% practicaban sexo anal ^21^ ; en Corea, el 34,0% eran mujeres, de las cuales el 47,3% tenían 50 años o más, edad donde la infección por VPH disminuye significativamente; además, el 70,0% no habían accedido a educación universitaria, y la proporción de hombres homo/bisexuales era del 66,2% ^23^ . En el estudio realizado en mujeres brasileñas, 52,2% de las participantes habían tenido sexo anal alguna vez en su vida ^31^ , mientras que en la presente investigación, todas las participantes cumplían con este criterio. En este mismo sentido, en el presente estudio, el porcentaje de hombres fue alto, la mayoría recibía terapia HAART, todos eran VIH positivos y tenían sexo anal como criterio de inclusión.

Hubo dificultades para comparar las frecuencias de infección por VPH halladas con los otros estudios, debido probablemente a las diferencias sociodemográficas y clínicas de los participantes, así como los criterios de inclusión en cada uno de los estudios. Asimismo, se pudo observar que la edad, el nivel educativo, las prácticas sexuales ano-receptivas, tener pareja sexual ocasional e iniciar vida sexual antes de la mayoría de edad son factores que predicen el riesgo de infección por VPH-AR. No obstante, el tiempo que llevan con el diagnóstico de VIH ^33^ se comportó como un factor protector y la terapia HAART ^22^ no fue significativa en el modelo estadístico aplicado. Por tanto, estas variables deberán ser evaluadas en futuros estudios a profundidad, debido a su implicación en la progresión a la NIA.

El tener pruebas que analizan los genotipos de otros VPH AR en conjunto no permite hacer un análisis de éstos por separado. Así, no se pudo tener el dato de la frecuencia de cada uno de ellos en la población analizada, lo cual daría mayor riqueza al estudio.

Al ser un estudio transversal, permitió hacer una exploración de los posibles factores de riesgo implicados en la infección anal por VPH oncogénico. Sin embargo, se requiere de estudios epidemiológicos analíticos que profundicen en el tema con el fin de detectar los aspectos críticos en los cuales se deben dirigir las campañas de prevención. El método de detección viral empleado no permitió discriminar entre los otros 12 tipos de VPH oncogénico. Por lo tanto, la prevalencia de co-infecciones puede ser mayor a la reportada debido a la posibilidad de existencia de más de un VPH de estos otros tipos en los participantes. Si bien el estado de la infección del VIH (Nivel de CD4+ y carga viral) son aspectos de gran importancia para la infección por VPH, no se disponían de datos actualizados suficientes para incluirlos en el análisis.

Los hallazgos de esta investigación permiten una línea base sobre la infección por VPH-AR en canal anal de la población con VIH, información hasta ahora desconocida en el contexto colombiano. La alta prevalencia para la infección por VPH-AR y en especial los tipos 16 y 18 evidencian la fuerte exposición que tiene la población VIH positiva con relaciones anales a estos genotipos de virus. Consecuentemente, apuntan para el riesgo de neoplasias anales y su posible progresión a cáncer anal; en especial si se tiene en cuenta las infecciones por múltiples genotipos, que sugieren persistencia de éstos por periodos prolongados, incrementando en mayor medida el riesgo ^22^ . La fuerte presencia de otros tipos oncogénicos del virus tales como 31, 33, 35, 39, 45, 51, 52, 56, 58, 59, 66 y 68, en el canal anal de estos pacientes revela la necesidad de profundizar en el papel que están desempeñando estos genotipos en la génesis del cáncer. Estos aspectos deben considerarse en las estrategias de prevención primaria que se evalúan actualmente para esta población.
